# The Role of Senescence-Associated Interferon in Age-Related Inflammation

**DOI:** 10.1093/geroni/igaf122.3332

**Published:** 2025-12-31

**Authors:** Mansi Shrivastava, Reema Banarjee, Bradley Olinger, Linna Cui, Amit Dey, Myriam Gorospe, Nathan Basisty

**Affiliations:** National Institute on Aging, Baltimore, Maryland, United States; National Institute on Aging, Bethesda, Maryland, United States; National Institute on Aging, Bethesda, Maryland, United States; National Institute on Aging, Bethesda, Maryland, United States; National Institute on Aging, Bethesda, Maryland, United States; National Institute on Aging, Bethesda, Maryland, United States; National Institute on Aging, Bethesda, Maryland, United States

## Abstract

Inflammaging, age-related deterioration of immune function, features persistent inflammation alongside an exacerbated immune response to pathogens. Senescent monocytes accumulate with age and release harmful proteins, potentially driving hyperinflammation. This study explores how senescent monocytes contribute to inflammaging and evaluates therapeutic interventions targeting these cells. Using an irradiation-induced senescence model in THP-1 monocytes, we analyzed the proteomic changes via data-independent acquisition (DIA) mass spectrometry. We assessed the impact of senescence on pathogen response by measuring LPS-induced inflammatory cytokines in senescent and non-senescent cells using a Mesoscale kit. Additionally, we tested various compounds for their ability to modulate inflammation and evaluated inflammatory signatures in monocytes. Finally, we examined proteomic profiles of circulating monocytes from 94 individuals aged 22-89 in the Genetic and Epigenetic Signatures of Translational Aging Laboratory Testing (GESTALT) study of aging to identify clinical associations between senescence and IFN signatures and aging phenotypes in vivo. Senescent THP-1 monocytes exhibited significant proteome remodeling, with increased 359 IFN-related proteins, including STAT1, OAS1, OAS2, MX1, and ISG15, while 1355 decreased proteins. Moreover, when stimulated with LPS, senescent monocytes exhibited a highly potentiated inflammatory cytokine response, including interleukins and IFN-ƴ, driving inflammaging and susceptibility. Notably, interventions targeting senescence or IFN signaling reduced these inflammatory responses. Further, the proteomic analysis of circulating monocytes in the GESTALT cohort showed that 27 senescence-associated proteins, correlated with CRP, were identified as potential biomarkers, highlighting systemic inflammation. These findings highlight senescence-associated IFN signaling as a key driver of inflammaging and suggest potential therapeutic strategies to mitigate age-related immune dysregulation.

